# Fluctuating Minds: Spontaneous Psychophysical Variability during Mind-Wandering

**DOI:** 10.1371/journal.pone.0147174

**Published:** 2016-02-10

**Authors:** Rodrigo A. Henríquez, Ana B. Chica, Pablo Billeke, Paolo Bartolomeo

**Affiliations:** 1 Inserm U 1127, F-75013, Paris, France; 2 Sorbonne Universités, UPMC Univ Paris 06, UMR S 1127, F-75013, Paris, France; 3 CNRS, UMR 7225, F-75013, Paris, France; 4 Institut du Cerveau et de la Moelle épinière, ICM, F-75013, Paris, France; 5 Department of Experimental Psychology, and Brain, Mind, and Behaviour Research Center, University of Granada, Granada, Spain; 6 Research Center for Social Complexity, Faculty of Government, University of Desarrollo, Santiago, Chile; 7 Department of Psychology, Catholic University Milan, Milan, Italy; Leibniz Institute for Neurobiology, GERMANY

## Abstract

Mind-wandering is the occasional distraction we experience while performing a cognitive task. It arises without any external precedent, varies over time, and interferes with the processing of sensory information. Here, we asked whether the transition from the on-task state to mind-wandering is a gradual process or an abrupt event. We developed a new experimental approach, based on the continuous, online assessment of individual psychophysical performance. Probe questions were asked whenever response times (RTs) exceeded 2 standard deviations from the participant’s average RT. Results showed that mind-wandering reports were generally preceded by slower RTs, as compared to trials preceding on-task reports. Mind-wandering episodes could be reliably predicted from the response time difference between the last and the second-to-last trials. Thus, mind-wandering reports follow an abrupt increase in behavioral variability, lasting between 2.5 and 10 seconds.

## Introduction

Mind-wandering, the occasional distraction we often experience while performing a cognitive task, is a self-generated condition, because it arises without any external precedent, varies over time spontaneously, and often interferes with the online processing of sensory information [1,2,3]. The very existence of mind-wandering supports the view that perception depends not only on its inputs, but also on the internal variability of the system.

Studies have addressed mind-wandering using different experimental definitions, e.g. task-unrelated thoughts [4,5], stimulus-independent thoughts [6], incidental self-processing [7], inner speech [8] momentary attentional lapses [9], or spontaneous thoughts [10,11]. Patterns of performance related to mind-wandering include variations in response times (RTs) during a sustained attention task [12], and increased response variability on a metronome response task [13,14]. Studies on the neural bases of mind wandering have described reduced amplitude of event-related potentials (ERPs) such as P300, MMN, and P2 components [1,15,16], as well as the activation of the brain default network [10,11]. A causal role of the dorsolateral prefrontal cortex has also been proposed [17].

Mind wandering is often explored by asking participants thought sampling questions (TSQs) on their state of mind, while they perform a sustained attention task [18]. However, this method is not optimal to assess whether the transition to mind-wandering is a process that develops gradually, or it is a unique event that triggers a global cognitive change. Moreover, the temporal rate of presentation of the TSQs can affect the likelihood of mind-wandering reports, because people are more likely to report mind-wandering as the time between TSQs increases [14].

In the present study, we aimed at defining some of the psychophysical conditions necessary for mind-wandering to occur. We used a new experimental approach, inspired by the classic sustained attention to response task (SART) [19]. We identified online RT outliers exceeding 2 SD from the participant’s mean RT. After each RT outlier, a TSQ was automatically asked. In comparison to traditional methods, this procedure allowed us to fit the behavioral variability associated with mind-wandering to the online statement of its occurrence in a more dynamic and ecological way.

## Materials and Methods

### Participants

Thirty-three healthy undergraduates (3 males) from the University of Granada participated in this study for course credit. A further participant was not considered in the analysis because he did not produce consistent responses, by often changing the response key assignment for targets and nontarget. All participants were right-handed, with their age ranging from 18 to 30 years (M = 23.71). Participants had normal or corrected to normal vision. They had no history of neurological disease and were free from psychoactive medication use. All participants provided their written informed consent prior to participating in the study. The local ethics committee from the University of Granada approved the experimental protocol.

### Stimuli

E-Prime^®^ software (version 2.1, Psychology Software Tools Inc., http://www.pstnet.com) was used to control stimuli presentation and response collection. Stimuli were displayed on a 21” monitor with a refresh rate of 60 Hz, situated at approximately 57 cm from the participants’ eyes. Ten randomly chosen upper-case letters were presented in rapid visual serial presentation. All the letters appeared inside an empty central white rectangle on a grey background. Each letter was presented for 100 ms, with an inter-stimulus interval varying randomly between 2,500 and 4,000 ms. There were three blocks of trials, each composed of 329 letters, and lasting for about 30 minutes. Participants were allowed to rest as much as they wanted between blocks.

### Procedure

**Fig 1:**
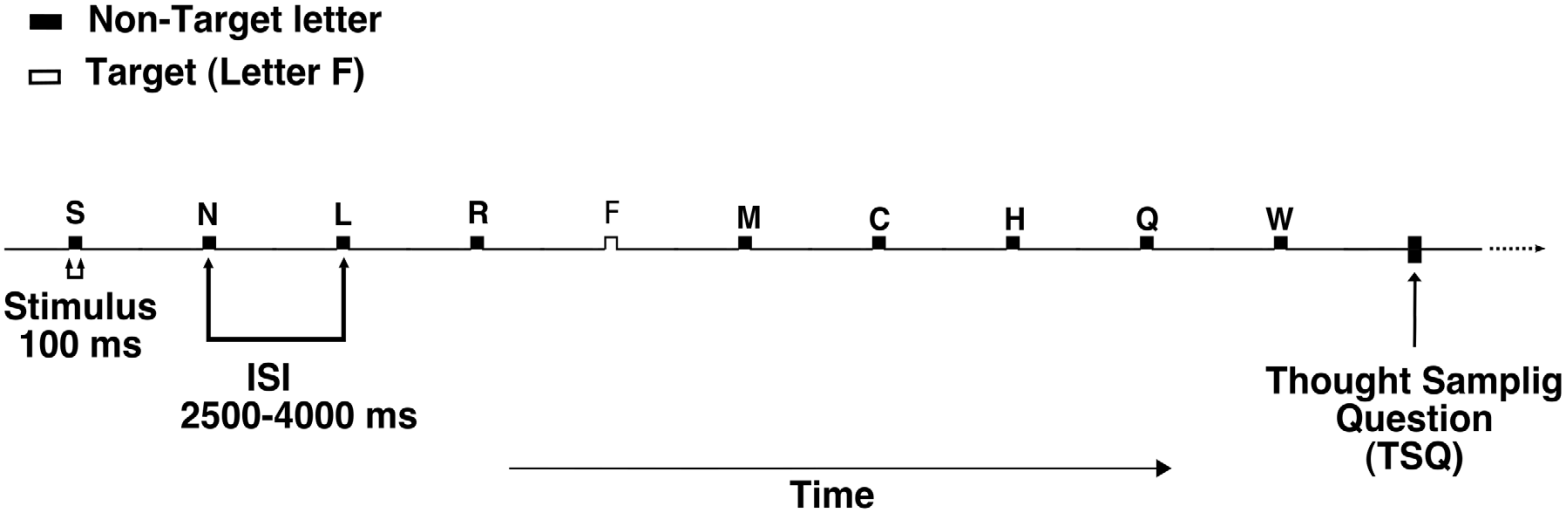
Schematic depiction of the experimental paradigm.

A keyboard with numeric keypad on the right side was used to provide responses. Participants were instructed to press as fast as possible a colored key (either a red “7” or a green “8”, in different participants) on the numeric keypad each time they saw the target (letter F) appearing. This condition occurred in 10% of the trials (Fig 1). In the remaining trials, when letters different from F appeared, they had to press the other colored key. The key assignment to target and nontarget letters was counterbalanced across participants. All participants used their right hand to respond. Mean RTs for nontargets were calculated online based on the last 5 responses. Whenever a RT exceeded 2 SDs from each participant’s RT mean within the last 5 trials (see below), the response was defined as an outlier RT, and a thought sampling question (TSQ) automatically appeared on the test display. Thus, average RTs before the TSQ were calculated on a number of consecutive trials, which could range from a minimum of 5 trials to a maximum of 10 trials (if no RT outlier was detected before trial 10). If no outlier RTs were produced during trials 6 to 9, after trial 10 a control TSQ appeared (“Standard TSQ”).

Each participant received an average of 48 TSQs per block. TSQs were formulated as follows: “Just before this question, your attention was distracted by.” Five possible responses were then proposed: (1) My attention was not distracted. (2) External distraction (e.g., uncomfortable posture, itches, sneezing, coughing). (3) Internal distraction (memories, imagination, thoughts). (4) Loosing vigilance (feeling of falling asleep). (5) Others. Participants had to respond by pressing a button corresponding to the number of the chosen alternative over the numeric keypad (respectively, keys 1, 2, 3, 4, or 5) (Fig 1). Response 1 (no perceived distraction) was considered as evidence that participants were on-task, while response 3 (internal distraction) defined mind-wandering (off-task condition).

## Results

The level of statistical significance was set at alpha = .05. Bonferroni correction was used to correct for multiple comparisons. Performance was generally accurate, with 86% correct performance for nontarget stimuli (which constituted 90% of the total stimuli), and 81% correct responses to target stimuli (10% of the total stimuli). Participants chose response 1 (on-task) 55.5% of the time, and response 3 (mind-wandering) 18.6% of the time (Table 1). A t-test performed on the arcsin-transformed response rates demonstrated that on-task responses were more frequent than off-task responses, *t* (32) = 6.87, *p* <0.001, *d* = 1.19, 95% CIs [.18, .34]. The vast majority (67.3%) of these on-task responses followed standard RTs (within 2 SD around the mean, see Procedure); 23.4% of all on-task responses were given after slow RTs (>2SD above the mean), and 9.4% were given for faster responses (>2SD below the mean).

**Table 1:** Percentages of response types.

**On-task**	55.5%
**External distraction**	14.5%
**Internal distraction (Off-task)**	18.6%
**Falling asleep**	8.8%
**Other**	2.6%

Responses to TSQs fell into two categories: responses related to outlier RTs (slow or fast), and responses related to standard (non-outlier) RTs. Based on previous evidence that RT variability is maximal in the trials just before mind-wandering episodes [13,20,21], we analyzed the 5 trials preceding on-task or off-task responses to TSQs (remember that 5 trials was the minimal possible interval between two TSQs).

**Fig 2:**
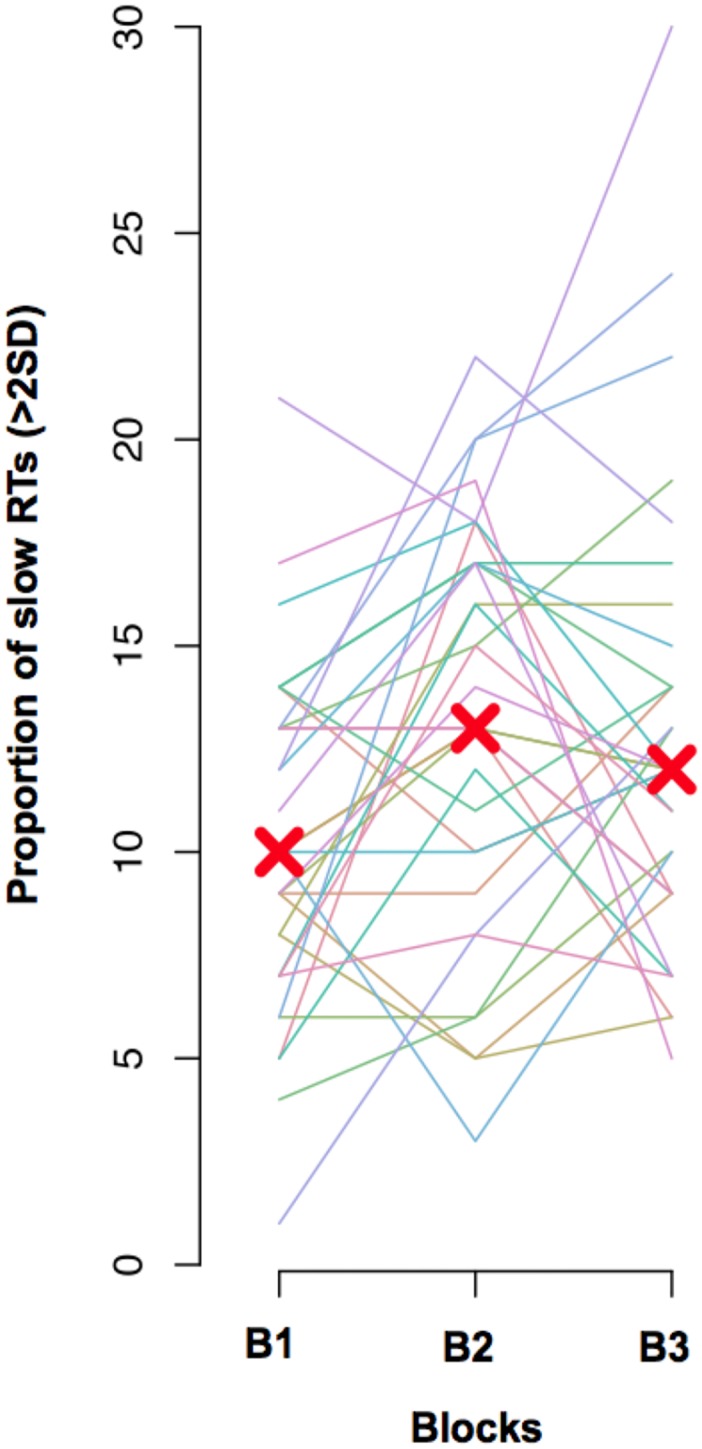
Distribution of slow RTs (> 2SD) across blocks. Each line represents a subject. Red Xs represent median percentages per block.

Fig 2 shows the distribution of “slow” RTs (> 2 SD above the mean) across blocks. Each line represents a subject; red Xs represent median percentages per block. Slow RTs were not uniformly distributed among blocks (Block 1, 10%; Block 2, 14.5%, Block 3, 12%; Friedman chi-squared = 12.28, df = 2, p = 0.002), because they increased in Block 2 compared with Block 1 (Friedman test, p = 0.007; Bonferroni correction for 3 comparisons yields an alpha value of 0.017). There was no significant difference between Block 2 and Block 3 (p = 0.7) or between Block 1 and Block 3 (p = 0.045). However, the percentage of off-task responses did not vary across blocks (Block 1, 13%; Block 2, 16%, Block 3, 15%; Friedman chi-squared = 4.06, df = 2, p = 0.13). Thus, while there may be some general tendency to an RT increase across blocks, perhaps resulting from fatigue, the rate of mind-wandering reports did not significantly vary along the experimental session.

**Fig 3:**
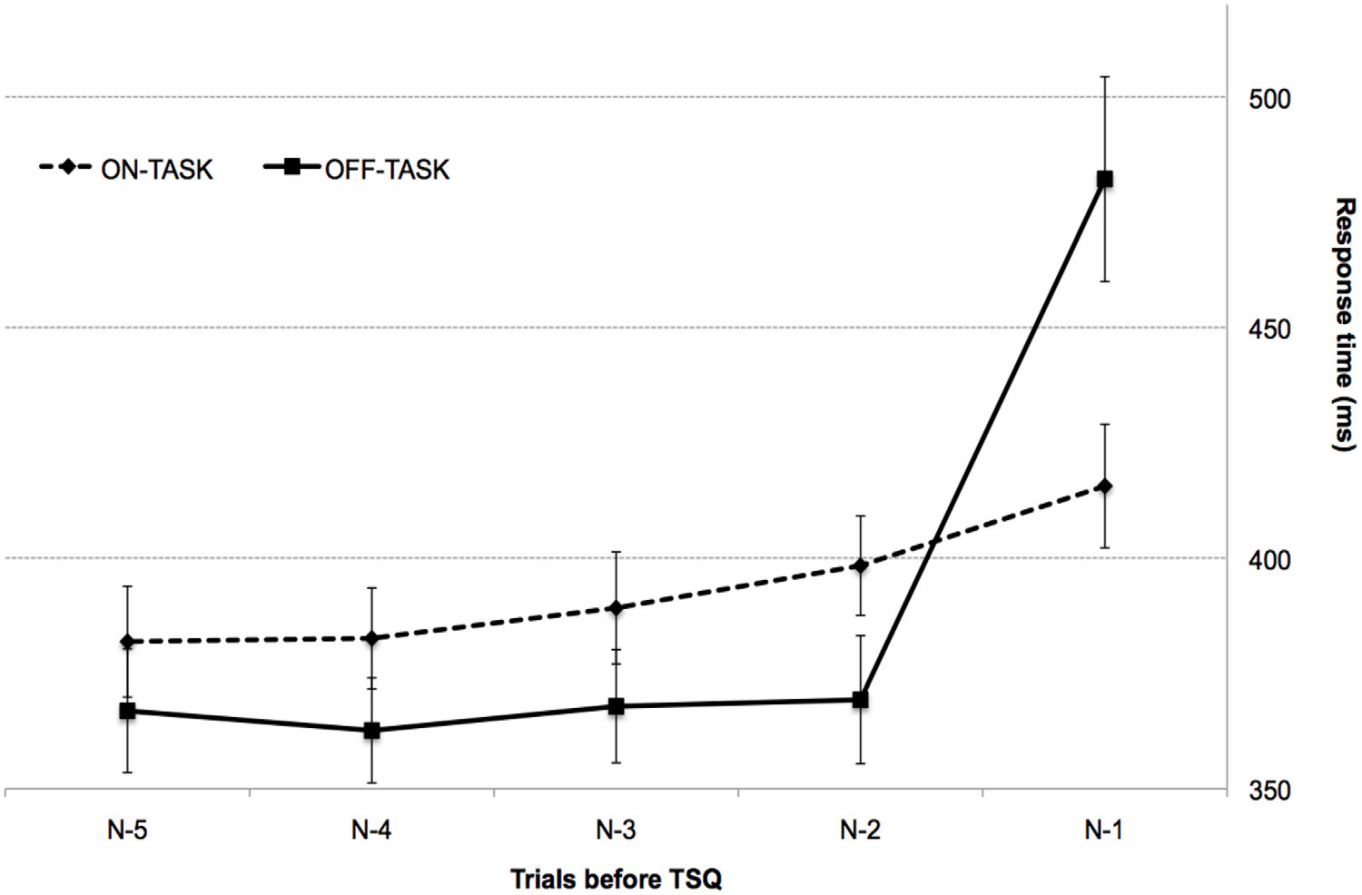
Distribution of mean RTs for the five trials before TSQ. Continuous line, off-task episodes related to mind-wandering; dashed line, on-task episodes. Error bars represent 1 standard error of the mean.

Fig 3 shows the evolution of RT during the last 5 trials before the TSQ. A 2x5 repeated measures ANOVA with condition (on-task and off-task) and trial position before the TSQ (N-1, N-2, N-3, N-4 and N-5) as within-participant factors revealed a main effect of trial position, F (4,128) = 44.00, p< .01, η_p_^2^ = .57, which interacted with condition, F (4,128) = 16.49, p < .01, η_p_^2^ = .34.

On-task and off-task conditions in trial N-5 led to similar RTs (Fig 3), *t*(32) = 1.86, *p* >.05, *d* = .32, 95% CIs [-1.39, 31.36]. In the following three trials (N-4 to N-2), RTs were on average 23-ms faster in the off-task condition compared with the on-task condition (all *ts*(32) > 2.84, all *ps* < .01, *d* = .49, 95% CIs [14.02, 44.05; 6.04, 36.52; 6.89, 32.98], for all comparisons). However, this pattern reversed in trial N-1 (just before the TSQ), which elicited 66-ms slower RTs on the off-task condition than on the on-task condition, *t*(32) = -3.53, *p* = 0.001, *d* = -.61, 95% CIs [-104.94, -28.18] (all these comparisons were Bonferroni corrected for 5 comparisons, with alpha = 0.01).

Although our aim was to explore internal distraction (mind wandering), we also assessed the RT profile of external distraction responses. We performed a 3x5 repeated measures ANOVA with response type (on-task, off-task task, and external distraction) and trial position before the TSQ (N-1, N-2, N-3, N-4 and N-5) as factors. The within-participant factors revealed a main effect of trial position, F (4, 128) = 96.22, p < .01, ηp2 = .75, which interacted with the response, F (4, 128) = 11.02, p < .01, ηp2 = .25. As shown in Table 2 and in S1 Fig, this interaction stemmed from the fact that on-task and external distraction RTs were similar for trials N-2 to N-5 (all ts(32) < .56, all ps > .57, d = -.09, 95% CIs [-18.31, 29.18; -15.06, 26.44; 6.89, 32.98; −23.88, 16.17]; Bonferroni correction for 5 comparisons, alpha = 0.01); however, similar to off-task responses, external distraction responses differed sharply from on-task responses for trial N-1, which elicited 108-ms slower RTs for external distraction responses than for on-task responses, t(32) = -6.09, p < .001, d = -1.05, 95% CIs [-145.47, -72.27]. Thus, off-task and external distraction responses gave rise to a similar RT profile.

**Table 2:** Mean RT and standard deviation for trials N-1 to N-5 before the TSQ for each possible response (on-task, off-task, external distraction, falling asleep, and other).

	N-1	N-2	N-3	N-4	N-5
**On-task**					
** Mean**	415.61	398.33	389.16	382.57	381.87
** SD**	77.55	62.34	70.33	63.25	69.44
**External distraction**					
** Mean**	524.48	392.89	383.47	387.65	385.72
** SD**	114.92	90.12	86.97	77.17	69.39
**Off-task**					
** Mean**	482.16	369.29	367.88	362.63	366.89
** SD**	128.51	80.21	70.89	65.74	76.55
**Falling asleep**					
** Mean**	323.79	362.67	371.73	368.24	360.16
** SD**	76.78	111.58	107.95	92.98	93.83
**Other**					
** Mean**	363.56	494.26	391.20	417.65	405.72
** SD**	154.13	310.48	112.57	223.81	135.61

We also assessed whether the mean RT for each of the five trials preceding a TSQ could predict the participant’s response (“on-task” or “off-task”), by performing a logistic regression analysis on the mean RT of each preceding trial (from N-1 to N-5). Results showed that increasing RTs significantly predicted reports of mind-wandering. The test of model coefficients was statistically significant, indicating that the predictors as a set reliably distinguished between trial position N-1 and N-2 (chi square = 34.09, *p* < .01 with df = 2). The Wald criterion demonstrated that trial N-2 and N-1 made a significant contribution to the prediction (*p* < .01). This is confirmed by Nagelkerke R square of .54 on second step that indicated a strong relationship between on- and off-task prediction and the position trials N-2 and N-1. Overall prediction success was 75.8% for the second step compared with less than 57.6% for the first step, which included only trial N-1 (Table 3).

**Table 3:** Logistic regression analysis of off-task and on-task condition for the last five trials before the TSQ.

	Variables	β	S.E	Wald	df	Sig.	Exp(β)
**Step 1.**	**N-1**	.006	.003	5.44	1	.020	1.0
	**Constant**	-2.79	1.21	5.26	1	.022	.06
**Step 2.**	**N-1**	.035	.010	12.30	1	.000	1.03
	**N-2**	-.045	.013	12.73	1	.000	.95
	**Constant**	2.06	1.76	1.37	1	.24	7.88

RTs for the other tested trial positions (from N-3 to N-5) failed to predict the participant’s response (all *p*s > .1). Finally, the RT difference between N-1 and N-2 trial significantly predicted participants’ responses to the TSQ (paired Wilcoxon signed rank test, Z = -4.51, *p* < .01, r = -0.57). Thus, not only was mind-wandering indexed by slow RTs to the last trial before the TSQ, but also, and more accurately, by the RT difference between the last and the last-but-one trial.

**Fig 4:**
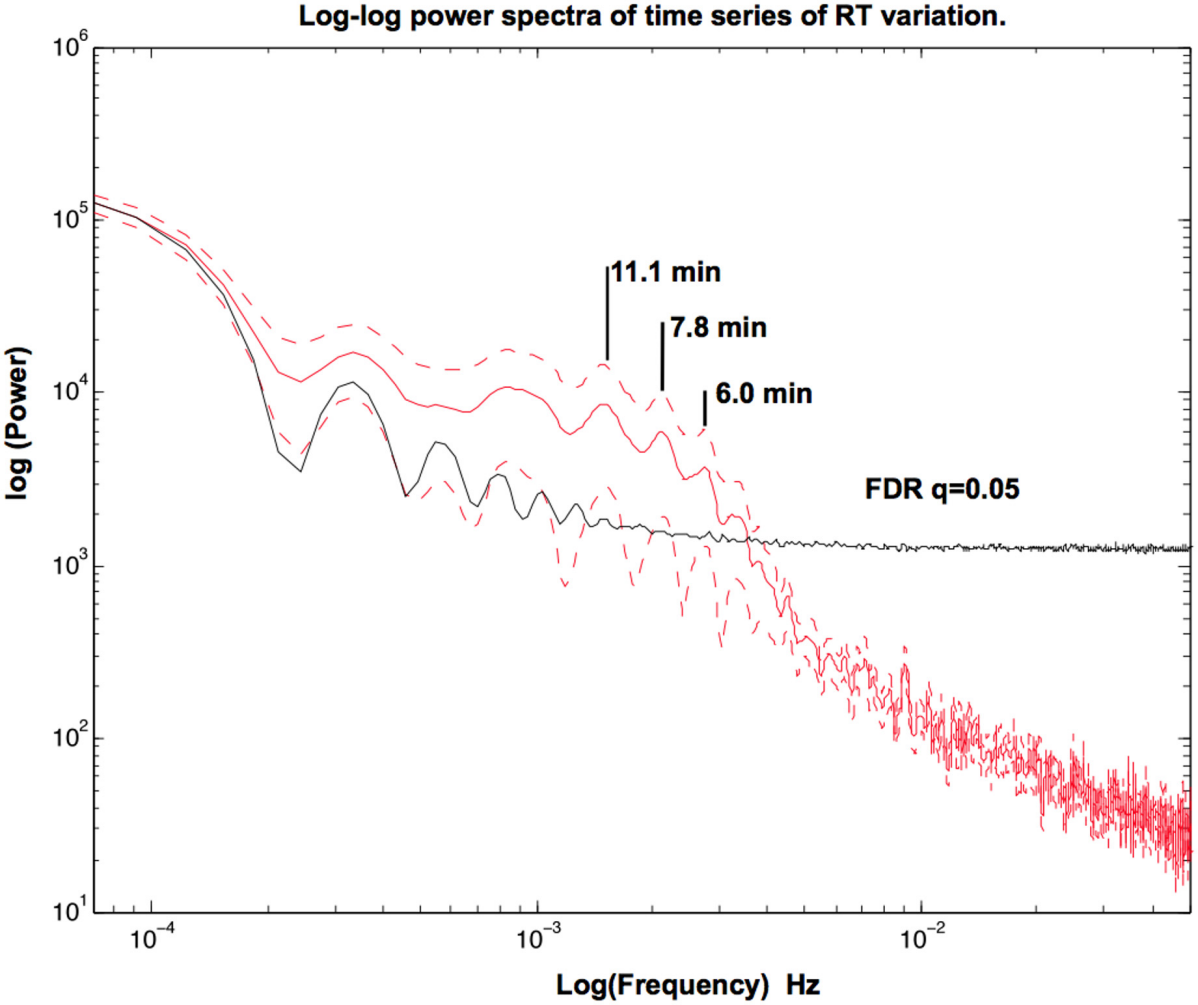
Log-log power spectra of time series of RT variation. Red continuous line: mean of the power spectrum across participants. Red dotted lines: standard error of mean. The black line represents the statistical threshold (Permutation test and false discovery rate (FDR), q = 0.05). The figure also shows the three only significant peaks at 11.1, 7.8, and 6.0 min.

In order to assess for long-range variations in RTs, we carried out a Fast Fourier Transform (FFT) to explore the variation of RTs through the task for each participant [22]. Then, we compared the resulting power spectrum with random distributions of the same data (2,000 permutations). RTs followed a 1/f distribution. Long-range variations (oscillations slower than one cycle per 6-minute period, Fig 4) were significantly greater that the random distribution. These oscillations represent variations longer than the maximum time interval between two TQS (41s). Finally, we assessed the possible relationship between these slow RT oscillations and the rate of off-task responses. We carried out correlation analyses between the rate of off-task responses and the power of these oscillations in 0.2-min bandwidth windows around the three fastest peaks in the FFT (11.1 min, 7.8 min, 6.0 min, see Fig 4). Results showed that subjects with greater power in the 5-min oscillation tended to produce less off-task responses (Spearman Correlation, 5.9–6.1. min, rho = -0.38, p = 0.02), while no correlation emerged for the other oscillations (7.9–7.7 min, rho = 0.08, p = 0.6; 11.0–11.2 min, rho = 0.27, p = 0.2).

## Discussion

A novel experimental procedure allowed us to identify episodes of mind-wandering based on the on-line assessment of RT fluctuations during a sustained attention task. Our procedure automatically detected outlier RTs and consequently triggered a TSQ during task performance. We explored different sources of mind-wandering based on individual responses to the five trials preceding the TSQ in both on- and off-task conditions. We observed that off-task reports were generally preceded by slower RTs as compared with trials before on-task responses. Note, however, that a substantial proportion of slow RTs (23%) occurred before “on-task” reports. Moreover, RTs tended to increase across blocks, without significant variation of the percentage of off-task responses. Thus, RTs alone are not to be considered as a reliable index of mind wandering. We also observed a clear difference between RTs in N-1 and N-2 trials compared to the remaining trials. This suggests that mind-wandering is not an attentional *state* in itself, characterized by a global slowing of responses. Rather, mind-wandering seems best characterized in a more dynamical way as a *transition* between different attentional states. Assessment of 1/f patterns showed the presence of slow oscillations (over several minutes) in RT performance. However, these oscillations either had no correlation or were negatively correlated with the rate of off-task responses. Thus, mind-wandering states seemed to interrupt these slow oscillations, rather than being part of them. Thus, our results suggest that mind-wandering is a local phenomenon lasting between 2.5 and 10 seconds, presumably driven by specific cognitive processes such as spontaneous fluctuations in the alertness system [23].

Previous studies have adjusted the presentation of evaluations of mind-wandering by using a pre-established rate of TSQs [18]. Our online method allowed us to automatically detect and assess different mind-wandering states continuously, and presumably in a more ecological way. Our psychophysical evidence demonstrates how dynamical local changes in the attentional system level can trigger global cognitive changes [24], and supports the hypothesis that changes of the attentional state not only depend on the nature of the stimuli, such as their physical properties, but also on the variability of the system itself [25–27]. Our approach could therefore inspire strategies to prevent inattention when mind-wandering can negatively impact cognitive performance [28].

Despite its clear and straightforward findings, this study has limitations. First, the observed RT profile was not able to clearly distinguish between internal and external distraction. Second, as compared to other methods our samples are presumably more specific, but also reduced in number. Third, it is difficult to estimate the potentially disruptive effects of the TSQs on the sustained attention task. Finally, the dichotomy between on- and off-task conditions might not be subtle enough to capture the richness of phenomenological experience. Thus, it is possible that this first attempt to make an online estimation of mind-wandering did not fully capture the richness of mind-wandering content, because our approach did not make any a priori assumptions about the cognitive definition of this phenomenon. Further experiments including a more precise evaluation of the cognitive content of mind-wandering should address these issues. To conclude, our new approach allowed us to identify mind-wandering as a dynamic transition between attentional states, which results in abruptly decoupling the attentional system from the external world.

## Supporting Information

S1 Dataset (ZIP)

S1 Fig Distribution of mean RTs for the five trials before TSQ. Continuous black line, off-task episodes related to mind-wandering; continuous grey line, external distraction episodes and black dashed line, on-task episodes. Error bars represent 1 standard error of the mean.
(TIFF)
