# Supplementary figures and images for: Fluctuating Minds: Spontaneous Psychophysical Variability during Mind-Wandering
Source: PLoS One. 2016 Feb 10;11(2):e0147174. doi: 10.1371/journal.pone.0147174 (PMC4749381; doi:10.1371/journal.pone.0147174)

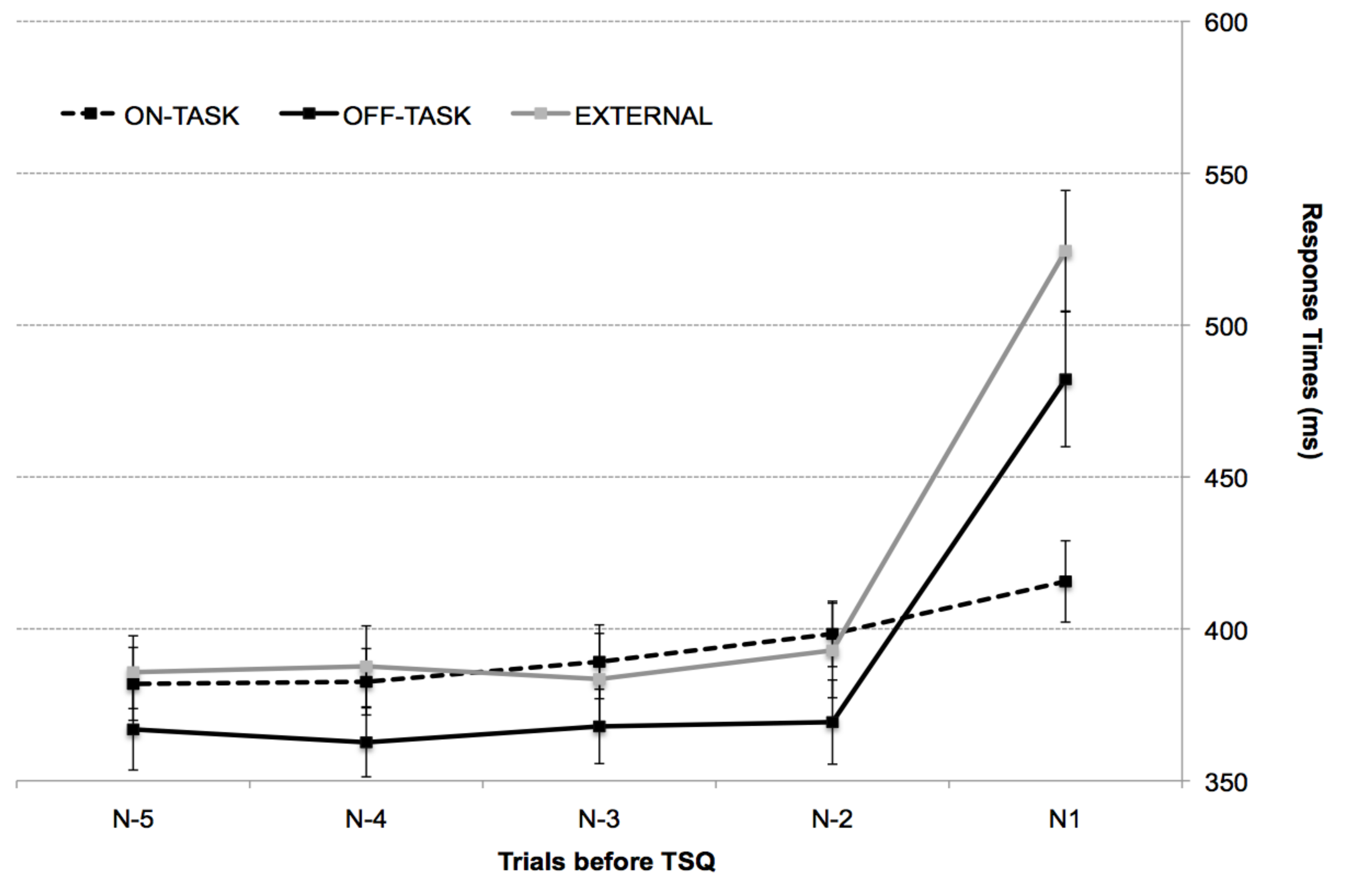

Supplement: S1 Fig — Continuous black line, off-task episodes related to mind-wandering; continuous grey line, external distraction episodes and black dashed line, on-task episodes. Error bars represent 1 standard error of the mean. (TIFF) [file pone.0147174.s002.tiff]
